# Dissecting entrepreneurial team research: a bibliometric analysis

**DOI:** 10.1007/s11846-023-00652-x

**Published:** 2023-03-24

**Authors:** Tin Horvatinovic, Mihaela Mikic, Marina Dabić

**Affiliations:** 1 Faculty of Business and Economics, University of Zagreb, Trg J. F. Kennedyja 6, Zagreb, 10 000 Croatia; 2 University of Dubrovnik, Dubrovnik, 20 000 Croatia; 3 School of Economics and Business, University of Ljubljana, Kardeljeva ploščad 17, Ljubljana, 1 000 Slovenia

**Keywords:** Entrepreneurial teams, New venture teams, Start-up teams, Bibliometric analysis, L26

## Abstract

Despite the massive volume of published articles, the pool of knowledge on entrepreneurial teams needs to be algorithmically classified and meticulously scrutinised. It is crucial for the field to be historically positioned under relevant themes, internally connected in terms of conceptual foundations, and systematically categorised in consonance with previously utilised frameworks of analysis. These concerns are resolved in this study by conducting a bibliometric analysis of 672 relevant articles. This form of analysis has not been previously employed on the topic of entrepreneurial teams. First, this study identifies eight main thematic clusters in the entrepreneurial teams field and their sub-themes. The eight main thematic clusters are: (i) *Intellectual Capital*, (ii) *Cognition and Behaviour*, (iii) *Science and Technology*, (iv) *Finance*, (v) *Transformation*, (vi) *Internationalisation*, (vii) *Family*, and (viii) *Community and Surroundings*. Second, the study reveals the clusters most needing restoration, relations between clusters, and input-mediator-output variables by their respective cluster. In addition, an implied scholarly depiction of entrepreneurial teams is articulated, which can serve as a basis for developing an *entrepreneurial teams theory*. Finally, promising avenues for future research are suggested for the entire field and every cluster specifically.

## Introduction

Entrepreneurs rarely establish and manage ventures alone. Instead, they are frequently part of a group of people, often labelled as entrepreneurial teams. Busenitz et al. (2003) recognised this fact almost two decades ago when they examined the structure of early research on entrepreneurship. In that article, they uncovered four distinct domains of inquiry at the core of entrepreneurship research: (i) entrepreneurial individuals and teams, (ii) mode of organising, (iii) opportunities, and (iv) environments. They postulated that future entrepreneurial research would develop at the intersection of those domains, a prediction that came to fruition further down the line (Busenitz et al. 2014). Therefore, the field of entrepreneurial teams has always been and continues to be at the heart of entrepreneurship research.

Given the importance and value of entrepreneurial teams research for entrepreneurship studies, it is not surprising to find numerous published articles, covering a vast array of subtopics. Prominently those include Ruef’s (2002) study on predisposition of creative action and Clarysse and Moray’s (2004) study on structuring of learning activities, among others. This landscape of knowledge on entrepreneurial teams was first encapsulated by Klotz et al. (2014) by adopting the acclaimed Input-Mediator-Output framework (IMO framework). According to them, empirical examinations (i) primarily used upper echelons theory as their theoretical background, (ii) zeroed in on a few conceptually distinct factors that are reported to be the main driving force behind the functioning of entrepreneurial teams, and (iii) lacked a focus on variables that channel those factors into beneficial outcomes. Klotz et al. (2014) also pointed to the diverging results of the effects demographic and social characteristics have on firms run by entrepreneurial teams. A meta-analysis by Jin et al. (2017) clarified such issues by showing that, from an inclusive perspective, experience and heterogeneity levels are advantageous for entrepreneurial teams.

Not all authors took the same route as Klotz et al. (2014), who applied a holistic view of the entire endeavour of entrepreneurial team research. Rather, other scholars selected a specific topic in the field and succeeded in developing a theoretical model, based on literature review tools, that stems from a large number of findings that comprised their sample. Examples include entrepreneurial team’s cognition (de Mol et al. 2015) and entrepreneurial team’s formation (Lazar et al. 2020). Thus, the arena of published studies on entrepreneurial teams is broad.

There are however, gaps and issues in the literature. Firstly, there is the scattered nature of published articles, meaning that they delve into unconnected subject matters, making it problematic to position them in a coherent conceptual framework. The innovative work of Klotz et al. (2014) paved the way for comprehending the results of a divided field. Later studies continued this tradition by arranging scientific findings according to stages in the development of entrepreneurial teams (Das et al. 2021) and their shared motifs (Knight et al. 2020). Despite those efforts, there is a growing need for rigorously and measurably identifying discrete collections of articles (with their standard postulates and themes) and displaying their connections. Secondly, since the research on entrepreneurial teams dates back to at least the early 1990s (Klotz et al. 2014; Knight et al. 2020), structuring publications across time to identify foundational and emerging themes is warranted. Finally, studies have yet to report on and categorise specific results of entrepreneurial teams publications according to the overarching theme to which they belong while respecting team variable taxonomy models.

Resolving these three issues is the focus of this study, which is achieved through the bibliometric analysis method.

The argument for the application of bibliometric analysis lies in the type of concerns identified in the field of entrepreneurial teams. First, specific topics are best identified by unbiased quantitative assessments of the whole publication spectrum, available through bibliometric analysis (Donthu et al. 2021). Second, the dispersion of research focus in scientific inquiries, present in entrepreneurial teams research, is best controlled under the postulates of bibliometric analysis (Donthu et al. 2021). Third, the results of bibliometric thematic clustering are the best starting points for further probing into the chronology, connectedness, and classification of entrepreneurial teams matters.

Apart from the intrinsic reasons, bibliometric analysis was selected to align entrepreneurial team research with recent literature review trends in business economics. Authors have used bibliometric analyses to analyse the publication structures of high-impact journals (e.g. Donthu et al., 2020; Mas-Tur et al., 2020) and specific subject matters such as absorptive capacity (Apriliyanti and Alon 2017), digital transformations (Shi et al. 2022), and the aftermath of Covid-19 (Verma and Gustafsson 2020).

Entrepreneurial topics are no exception. Some authors took a bird’s-eye view of the entrepreneurial field (Ferreira et al. 2019), while others narrowed their focus. Foundational and emerging themes were uncovered, for instance, in SME internationalisation (Dabić et al. 2020a), business incubators (Deyanova et al. 2022), the business context of sporting activities (Huertas González-Serrano et al. 2020), crowdfunding campaigns (Gil-Gomez et al. 2021), managing complexities in the sharing economy (Kraus et al. 2020), and entrepreneurial ethical judgements (Vallaster et al. 2019). But, surprisingly, not to entrepreneurial teams.

Therefore, the bibliometric analysis is applied to high-impact papers collected from the Web of Science database. Understanding the importance of a consistent paradigm, this study opted for the definition of entrepreneurial teams proposed by Knight et al. (2020) to recognize the articles considered part of the field. Using those boundary points, the final sample size consisted of 672 articles.

Based on the compiled sample, eight underlying conceptual building blocks of entrepreneurial teams research are outlined in this study. These eight building blocks, or *clusters*, are: (i) *Intellectual Capital*, (ii) *Cognition and Behaviour*, (iii) *Science and Technology*, (iv) *Finance*, (v) *Transformation*, (vi) *Internationalisation*, (vii) *Family*, and (viii) *Community and Surroundings*. Each cluster is analysed to disclose the specific and predominant findings in all relevant thematic areas.

Such an itemised description of the field grants support for a more promising agenda for future research. This exposition of results also allows the incorporation of variables of interest to scholars in the IMO framework according to their cluster affiliation. Thus, part of this study is an extension of the founding paper by Klotz et al. (2014). Furthermore, by putting all eight clusters in chronological order, a temporal analysis can be made. Finally, the identified pattern of heavy usage of the upper echelons theory by Klotz et al. (2014) is expanded. Subsequently, it is demonstrated that the diversity of theoretical underpinnings and scholarly interpretation of entrepreneurial teams is broader than earlier assumed. This lack of unity in the theoretical foundations of the entrepreneurial teams field stifles the progress that could be made (Knight et al. 2020). Thus, this study proposes that the unification of the field, here designated as *entrepreneurial teams theory*, could commence from the discovered underlying latent theoretical consensus of researchers.

Finally, a bibliometric analysis permits recognising the most influential authors, papers, journals, institutions, and countries present in the research of entrepreneurial teams. The findings emanating from these considerations elude to the existence of geographical bias, on the country level, in the sampled articles.

Considering the design and execution of this study as described, the accepted and envisioned contributions of modern literature reviews are realised. That is, an overview of current research, the appraisal of obtained findings, and the avenues for further inquiries (Kraus et al. 2022) are all clearly expressed and elaborated for the field of entrepreneurial teams.

## Methodology

### Bibliometric analysis description

Bibliometric analysis is employed in this paper as a literature review device to investigate the scientific research on entrepreneurial teams. Such analysis was primarily devised to handle, in a quantitative way, large amounts of objective publicly available library data on published documents. In other words, bibliometric analysis encompasses a wide array of statistical means to provide an exhaustive overview and description of the selected topic (Donthu et al. 2021). Because bibliometric analysis relies on quantitative methods, it is reported to be more rigorous and less prone to various author biases compared to traditional narrative literature reviews (Gonzalez-Loureiro et al. 2015; Dabić et al., 2020 a). In addition, it can aid researchers in identifying germane studies and guide them to arrange their findings more succinctly (Linnenluecke et al. 2020). Furthermore, bibliometric tools today are more accessible due to software advancements and usable databases of scientific journals (Donthu et al. 2021).

Despite these benefits, bibliometric analysis is not a one-size-fits-all approach and should be utilised in specific circumstances. To fully exploit bibliometric analysis’s advantages, the topic should be broad in scope and scrutinised by many publications (Donthu et al. 2021). Concerning entrepreneurial teams, the first point was addressed in the introduction section, where many facets of entrepreneurial teams were outlined. The second point will be covered in the next section when the sample construction will be described in detail.

### Data collection and used methods

The first step in collecting the data was determining the appropriate database for published studies. Ordinarily, authors choose between the Web of Science database and the Scopus database for conducting a bibliometric analysis. For this study, the Web of Science database was chosen for three reasons. Firstly, while acknowledging the advancement of the Scopus database, the Web of Science database is still the prevailing scientific database regarding impact assessment (Zhu and Liu 2020). Second, the Scopus database could not capture some of the older publications on entrepreneurial teams since it was established a few years after the Web of Science database (Falagas et al. 2008). Lastly, using the Scopus database could potentially diverge the findings of this study from previous literature reviews on entrepreneurial teams (de Mol et al. 2015; Knight et al. 2020) since they utilised the Web of Science database to build their sample.

In the second step, search terms for topics were identified. In line with Knight et al. (2020), search terms and their respective variations amounted to “start-up team, new venture team, nascent team, founding team, entrepreneurial team, and prefounding team”. In this article, the term entrepreneurial teams is used to capture all the synonyms since it encapsulates the whole entrepreneurial process, instead of a certain stage in the development of the firm. This inquiry allowed the incorporation of a broad spectrum of publications since it yielded a result of 32,053 documents in March of 2022.

However, not all articles were selected for the final sample since some exclusion criteria were imposed. First, research areas not relevant to this study were excluded from the sample, such as those outside the entrepreneurial domain. Examples include sports or medical teams in hospitals. Second, documents categorised as book chapters and solely as proceeding papers were removed. This practice is not uncommon in bibliometric studies (e.g. Deyanova et al., 2022; Merigó et al., 2016) to capture the most pertinent documents. Third, articles that were published in the Social Sciences Citation Index (SSCI) were kept, while other papers were ruled out. Including only the most impactful journals for analyses stems from other literature reviews of entrepreneurial teams (de Mol et al. 2015; Klotz et al. 2014; Knight et al. 2020). Fourth, only English-written papers were considered.

After applying these exclusion criteria, each remaining article was checked for compatibility with the proposed definition of entrepreneurial teams by Knight et al. (2020). This definition states that an entrepreneurial team is a: “group of two or more people who work together interdependently to discover, evaluate, and exploit opportunities to create new products or services and who collectively have some ownership of equity, some autonomy of decision-making, and some entitativity” (Knight et al. 2020, p. 255). Based on that definition, most articles could be readily labelled as research on entrepreneurial teams or not. However, even though Knight et al. (2020) offer a precise definition, there were instances where author discretion must be applied. This issue was prevalent with articles in the psychology literature since sample characteristics, which are essential for this study, were often not described in detail. Therefore, such articles were further checked for the mentioning of entrepreneurial references. For the sake of accuracy, an article was removed from the sample when there was a considerable probability that the article did not fit the definition mentioned earlier.

Furthermore, articles that examined team functioning in a corporate setting were eliminated. In addition, articles that investigated solely the structure of teams employed by entrepreneurs, such as research and development teams, were likewise not considered. Also, studies that conceptualised entrepreneurial teams simply as a control variable without postulating or explaining the effect that those teams produced were ruled out from the sample. Similarly, literature reviews or conceptual articles that devoted little attention to entrepreneurial teams were omitted. Finally, studies that tested their hypotheses on a sample of students with real-world simulation problems were excluded even if they built up their hypothesis as if the students were operating as an entrepreneurial team. The reason for this criterion is the fact that it does not concord with the equity ownership part of the above-remarked definition of entrepreneurial teams.

After completing this process, the final step of data gathering consisted of inspecting the references in the leading literature reviews on entrepreneurial teams (de Mol et al. 2015; Klotz et al. 2014; Knight et al. 2020) to make sure that all relevant articles would be a part of the final sample. This described article selection mechanism yielded a final sample of 672 articles. Given that Donthu et al. (2021) recommend having more than 500 papers for conducting a bibliometric analysis, the sample size of 672 articles accumulated in this study is sufficient.

These articles were analysed through two main bibliometric approaches: (i) performance analysis and (ii) science mapping. Performance analysis consists of tools that help to delineate the contributions to the field in question from the author, article or publication point of view. More concretely, publication-related metrics, citation-related metrics, and citation-and-publication metrics were utilised (Donthu et al. 2021). Regarding science mapping, bibliographic coupling was carried out in the VOSviewer software. The bibliographic coupling technique presumes that articles are connected if they have a fair share of common references. It is used to uncover a broad range of themes that currently dominate the research field (Donthu et al. 2021).

## Results

### Performance analysis

A short sample description is provided in Table 1. The selected articles were published in 141 journals from 1990 to 2022. Such a large number of publication outlets indicates that entrepreneurial teams is a very eclectic topic. Furthermore, this field of research is highly influential, with an average of around 63 citations per article.

**Table 1:** General information of the sampled studies

Description	Results
Timespan	1990–2022
Documents	672
Average citations per documents	63.83
Average citations per year, per document	5.61
Author’s keywords	1,471
Authors	1,388
Single-authored documents	73
Documents per author	0.48

Source: compiled by authors

**Fig. 1:**
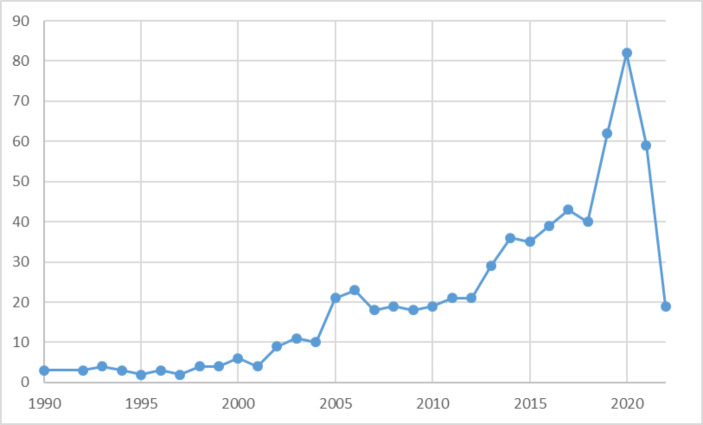
Publication dynamics of selected articles Source: compiled by authors

The number of publications on entrepreneurial teams steadily grew in the observed period but exhibited some differences in a few sub-periods. All years from 1990 to 2004 exhibited eleven or fewer publications, with an overall average of 4.86 publications per year during that time frame. Afterwards, from 2005 to 2012, the total number of publications increased, and there was almost an equally distributed number of articles, with an average of 20 published articles per year. The next period, from 2013 to 2020, was characterised by the amplification of research with an average of 47.75 published articles per year and ending in 82 published articles just in 2020. Although 2021 was still a very productive year with 59 articles, in comparison to 2020, the aggregate amount of articles decreased. Finally, 19 articles were published in 2022 by the time the sample was constructed for this study. Figure 1 graphically displays the above-described sequences.

Table 2 reveals the citation structure of the sampled articles. Only two papers accumulated more than 1,000 citations in the Web of Science database. The most cited article established a causal role of entrepreneurial teams in network formation (Eisenhardt and Schoonhoven 1996), while the second most cited article associated the characteristics of entrepreneurial teams with firm growth (Eisenhardt and Schoonhoven 1990). The following two classes of citations are empty. Relating to entrepreneurial teams, other highly influential articles explored the topics of social capital (Shane and Stuart 2002; Stam and Elfring 2008), entrepreneurial self-conceptualisation (Fauchart and Gruber 2011), legitimisation (Delmar and Shane 2004), financing practices (Ahlers et al. 2015; Baum and Silverman 2004), resource acquisition processes (Zott and Huy 2007), firm expansion activities (Reuber and Fischer 1997), and team-level competencies (Colombo and Grilli 2005). Not surprisingly, the largest number of articles had between 1 and 99 citations, while 52 had zero citations.

**Table 2:** Citation structure of papers

Total citations	Number of papers	Percentage of papers
≥ 1000	2	0.30%
900–999	0	0.00%
800–899	0	0.00%
700–799	1	0.15%
600–699	4	0.59%
500–599	2	0.30%
400–499	4	0.59%
300–399	12	1.79%
200–299	30	4.46%
100–199	63	9.38%
1–99	502	74.70%
0	52	7.74%
Total	672	100%

Source: compiled by authors

When local citations are also considered, a more detailed picture of the citation landscape is provided. Local citations refer only to citations made by articles in the collected sample. Eisenhardt and Schoonhoven’s (1990) article is the most locally cited, with 146 citations. Among the class of highly cited articles, the article by Beckman et al. (2007) has the highest ratio of local to total citations. One interpretation of this finding is that the article by Beckman et al. (2007) covers a broad range of topics that is of interest to entrepreneurial teams scholars. By contrast, Eisenhardt and Schoonhoven’s (1996) article, the most cited article in the Web of Science database, has a very low local-to-total citation ratio of 2.22%. This implies that Eisenhardt and Schoonhoven’s (1996) work is predominantly utilised in other fields of research, presumably the entrepreneurial networking literature. Apart from giving an overview of the field (Klotz et al. 2014) and the previously mentioned matter of team competencies (Colombo and Grilli 2005), other highly locally cited articles are related to the evolution of the team (Beckman and Burton 2008; Ucbasaran et al. 2003), conduct of the firm (Beckman 2006), configuration of the team (Amason et al. 2006; Ensley and Hmieleski 2005), and extent of union inside the team (Ensley et al. 2002).

**Table 3:** Most locally cited documents

Authors	Title	Journal	LC	LC/TC
Eisenhardt and Schoonhoven (1990)	Organizational Growth: Linking Founding Team, Strategy, Environment, and Growth Among U.S. Semiconductor Ventures, 1978–1988	*Administrative Science Quarterly*	146	12.92%
Klotz et al. (2014)	New Venture Teams: A Review of the Literature and Roadmap for Future Research	*Journal of Management*	97	35.14%
Beckman et al. (2007)	Early teams: The impact of team demography on VC financing and going public	*Journal of Business Venturing*	92	35.80%
Ensley et al. (2002)	Understanding the dynamics of new venture top management teams: cohesion, conflict, and new venture performance	*Journal of Business Venturing*	88	28.21%
Ucbasaran et al. (2003)	Entrepreneurial Founder Teams: Factors Associated with Member Entry and Exit	*Entrepreneurship Theory and Practice*	76	35.02%
Amason et al. (2006)	Newness and novelty: Relating top management team composition to new venture performance	*Journal of Business Venturing*	69	30.94%
Beckman (2006)	The Influence of Founding Team Company Affiliations on Firm Behavior	*Academy of Management Journal*	68	19.05%
Beckman and Burton (2008)	Founding the Future: Path Dependence in the Evolution of Top Management Teams from Founding to IPO	*Organization Science*	59	21.38%
Colombo and Grilli (2005)	Founders’ human capital and the growth of new technology-based firms: A competence-based view	*Research Policy*	55	10.09%
Ensley and Hmieleski (2005)	A comparative study of new venture top management team composition, dynamics and performance between university-based and independent start-ups	*Research Policy*	53	24.77%

Source: compiled by authors

Note: LC is local citation; TC is total citation.

Next, Table 4 provides a list of the most productive authors in the sampled articles. Mike Wright produced the most articles with 14, followed by Bart Clarysse and Michael D. Ensley with 11. Michael D. Ensley had the most citations per paper among the most productive authors.

**Table 4:** Most productive authors

Author	No. of papers	No. of total citations	Citations per paper
Wright M	14	2,185	156.07
Clarysse B	11	849	77.18
Ensley MD	11	2,220	201.82
Knockaert M	10	321	32.10
Breugst N	8	123	15.38
Busenitz LW	8	771	96.38
Hmieleski KM	8	1,213	151.63
Patzelt H	8	150	18.75
Gruber M	7	1,124	160.57
Lockett A	7	1428	204

Source: compiled by authors

Information regarding the statistics of publication outlets is displayed in Table 5. The *Journal of Business Venturing* published the highest number of papers, with 63. The second is *Small Business Economics* with 50, and the third is *Entrepreneurship Theory and Practice* with 42. In terms of citations, the *Journal of Business Venturing* is the most influential, with 7,273 total citations in the Web of Science database. *Entrepreneurship Theory and Practice* and *Academy of Management Journal* are second and third in the total number of citations.

**Table 5:** Most active journals measured in the number of papers and citations

Sources	No. of papers	Sources	No. of citations
*Journal of Business Venturing*	63	*Journal of Business Venturing*	7,273
*Small Business Economics*	50	*Entrepreneurship Theory and Practice*	3,653
*Entrepreneurship Theory and Practice*	40	*Academy of Management Journal*	3,312
*Journal of Small Business Management*	27	*Organization Science*	3,209
*Journal of Business Research*	24	*Research Policy*	2,918
*Research Policy*	22	*Administrative Science Quarterly*	2,700
*Strategic Entrepreneurship Journal*	21	*Small Business Economics*	2,063
*Academy of Management Journal*	20	*Strategic Management Journal*	1,425
*International Entrepreneurship and Management Journal*	20	*Management Science*	1,243
*Organization Science*	20	*Strategic Entrepreneurship Journal*	1,081

Source: compiled by authors

The predominance of management and business journals is expected, given the nature of the topic of entrepreneurial teams. Despite that fact, journals in other fields also played a role in publications on entrepreneurial teams. This is especially the case with psychology. *Frontiers in Psychology* is the journal that published the highest number of papers (12) in a pool of journals that predominantly deal with psychological topics.

Before proceeding to scientific mapping, it is worth noting the most influential countries and institutions published on entrepreneurial teams. The United States of America was undoubtedly the most productive country, with 327 papers and 28,186 citations. Regarding the number of papers, England is second, and the People’s Republic of China is third. Of the most influential countries, Canada has the most citations per document, with 86.40.

From the faculty perspective, the University of North Carolina has the highest number of published papers with 25, followed by Ghent University with 23, and the Technical University of Munich with 18. Stanford University has the most citations per document, with 263.94, a finding that is predominantly fuelled by the two most cited papers in the whole sample.

Further information on the country and institution-level production are displayed in Tables 6 and 7.

**Table 6:** Most influential countries

Country	No. of papers	No. of citations	Average citation per article
United States of America	327	28,186	86.20
England	99	6,171	62.33
People’s Republic of China	84	1,801	21.44
Germany	75	3,286	43.81
Italy	59	1,982	33.59
Spain	55	1,841	33.47
Netherlands	44	2,334	53.05
Canada	40	3,456	86.40
Belgium	36	1,536	42.67
Switzerland	34	1,800	52.94

Source: compiled by authors

**Table 7:** Most influential institutions

Institution	No. of papers	No. of citations	Average citation per article	
University of North Carolina	25	1,335	53.40	
Ghent University	23	1,379	59.96	
Technical University of Munich	18	639	35.50	
Stanford University	16	4,223	263.94	
University of Missouri System	16	1,390	86.88	
University of Nottingham	16	2,252	140.75	
University System of Georgia	16	1,665	104.06	
Imperial College London	14	832	59.43	
Indiana University System	14	876	62.57	
Texas Christian University	14	1,746	124.71	
University System of Maryland	14	2,204	157.43	

Source: compiled by authors

### Science mapping

**Fig. 2:**
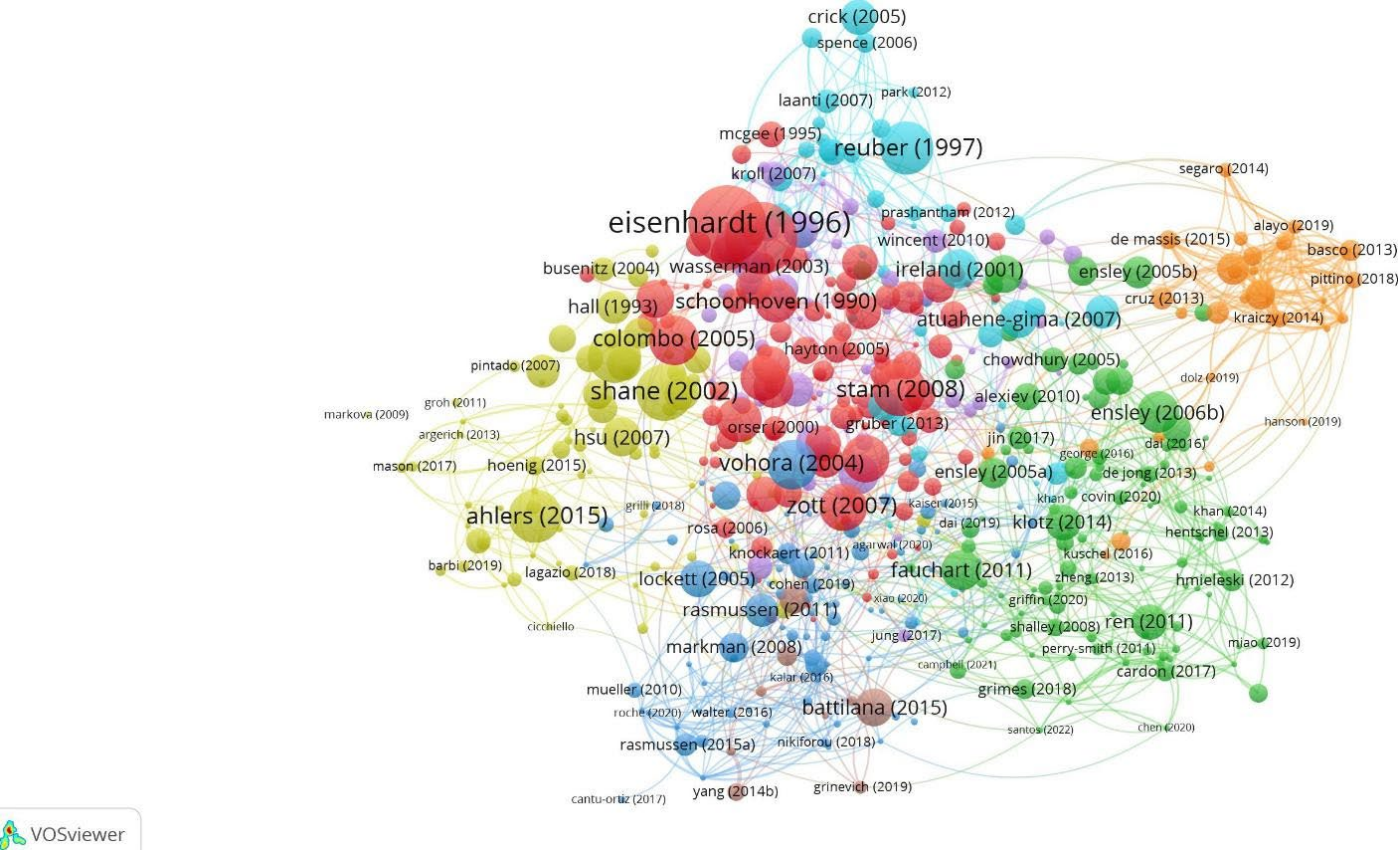
Bibliographic coupling Source: compiled by authors Notes: the cut-off point for article entry in bibliographic coupling was 3 citations; fractional counting was used

The following image, Fig. 2, is the result of the scientific mapping procedure.

**Fig. 3:**
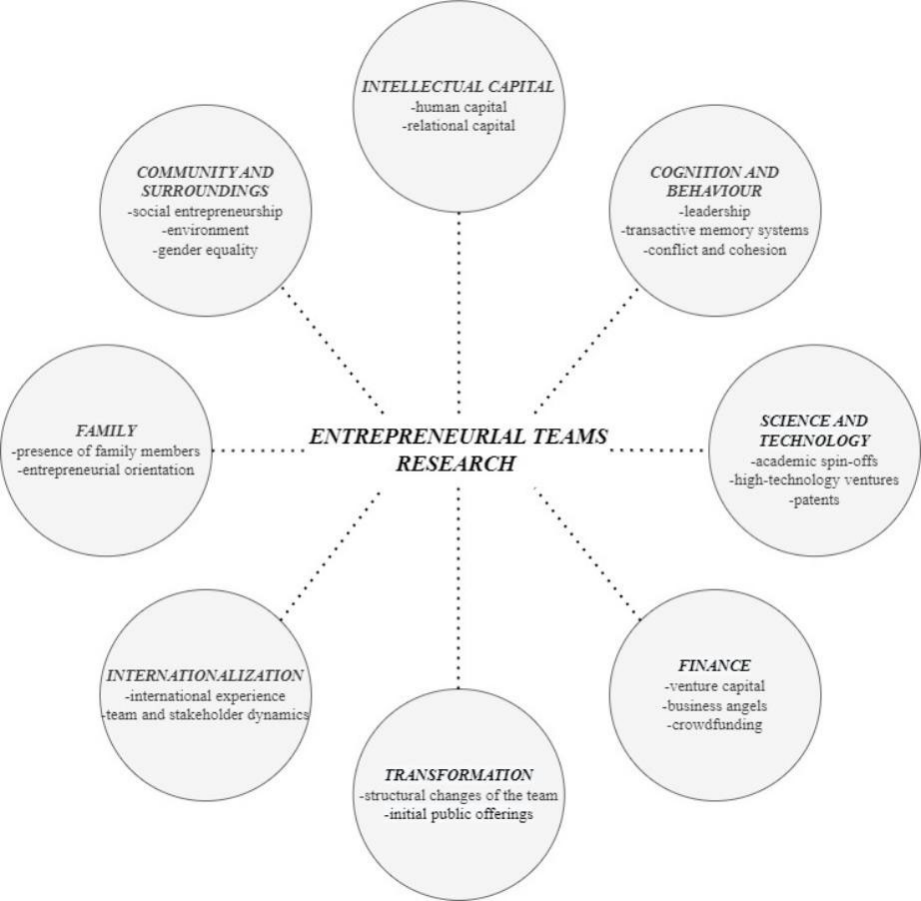
Summation of clusters and researched topics Source: compiled by authors

The results of the bibliographic coupling revealed eight distinct clusters, namely: (i) *Intellectual Capital*, (ii) *Cognition and Behaviour*, (iii) *Science and Technology*, (iv) *Finance*, (v) *Transformation*, (vi) *Internationalisation*, (vii) *Family*, and (viii) *Community and Surroundings*. All the uncovered clusters and highly represented subtopics inside the clusters are graphically displayed in Fig. 3.

These eight clusters, their respective subtopics, and their main findings are analysed below.

#### Red cluster (1)- Intellectual Capital

The first cluster that is analysed is the red cluster in Fig. 2. Most published material that constitutes this cluster falls under intellectual capital literature. More specifically, the two most represented themes are *human* and *relational capital*.

The *human capital* of entrepreneurial teams is the most central concept in this cluster. As a critical intangible resource of firms, human capital was studied from many angles, such as education levels (e.g. Colombo and Grilli, 2005; Watson et al., 2003) and team size (e.g. Eisenhardt and Schoonhoven, 1996; Ruef, 2002). One aspect which stands out among them, in terms of representation, is team members’ experience.

Joint work experience, industry experience and prior entrepreneurial experience are the three dominant types of experiences in this cluster. The findings of all three kinds of team members’ experience levels have an ambiguous impact on many firm-level outcomes, meaning that contradictory findings exist within the literature. Despite this claim, a general directionality can be established. For instance, several studies point to the perks of prior joint work experience (Eisenhardt and Schoonhoven 1990; Roure and Keeley 1990) and industry experience (Eisenhardt and Schoonhoven 1996; Oe and Mitsuhashi 2013) for firm-level outcomes. Startlingly, similar results are not present in the case with prior entrepreneurial experience, given that most scholars (Aspelund et al. 2005; Oe and Mitsuhashi 2013; Schoonhoven et al., 1990) cast doubt that it can help entrepreneurial teams better manage their firms.

*Relational capital* is the second form of intellectual capital that researchers examined. In terms of the number of studies, it received less attention than human capital. Nonetheless, interesting insights emerged from studies where networks of entrepreneurial teams were scrutinised. Positive effects of external networks manifest themselves if all team members are part of the network building and exploitation process (Neergaard 2005) and where the key roles of team members are defined (Grandi and Grimaldi 2003). These measures must be implemented from the beginning of the venture since initially established networks are crucial for resource procurement (Packalen 2007) and, subsequently, firm growth (Brinckmann and Hoegl 2011). If managed correctly as stipulated, entrepreneurial networks can lead to higher firm performance (Vissa and Chacar 2009) and innovation (Chen and Wang 2008).

#### Green cluster (2)- cognition and Behaviour

The green cluster reveals the predominant topics in behaviour and cognition. These include *leadership*, *transactive memory systems*, *conflict* and *cohesion*.

Research on *leadership* comprises a fair share of published papers in this cluster. The focus of authors that probed into the topic of leadership was to determine the effect leadership has on business and team performance. The presence of leadership behaviour that drives the exploration and exploitation of entrepreneurial opportunities inside entrepreneurial teams can positively influence team performance as a whole, as well as the performance of individuals that are part of the team (Miao et al. 2019). Another type of leadership, transactional leadership, is also beneficial to entrepreneurial teams. Leadership of this kind, which emphasizes rewards in guiding the behaviour of entrepreneurial teams, has a positive effect on business performance and will be more potent in environments with less uncertainty (Ensley et al. 2006).

The second theme research in this cluster has focused on is *transactive memory systems*. This concept of interdependence among team members, displayed in the mutual understanding of where the expertise lies within the team, has shown to be auspicious for entrepreneurial teams. High levels of development of transactive memory systems are linked to higher firm performance (Heavey and Simsek 2015; Zheng 2012), entrepreneurial orientation (Dai et al. 2016), team learning (Kollmann et al. 2020), and team identification (Kollmann et al. 2020).

Following up on the theme of cognitive aspects of entrepreneurial teams, it is unsurprising that the existence of *conflict* received attention from entrepreneurial scholars. What is somewhat surprising, however, are the mixed results of these studies. Some studies report many advantages that arise from conflict. For instance, a high task-related conflict between entrepreneurial team members can lead to superior entrepreneurial strategies (Li and Li 2009). Similarly, a task-related conflict between entrepreneurial teams and their funding providers results in better venture performance (de Jong et al. 2013; Higashide and Birley 2002). However, these conflicts can be detrimental to the teams’ effectiveness and efficiency (Khan et al. 2015). Contrary to team conflict, studies on *team cohesion* consistently display the positive impact it has on venture performance (Chen et al. 2017; Ensley et al. 2002).

#### Blue cluster (3)- science and technology

The third identified cluster in blue in Fig. 2 mainly consists of papers that look into the opportunities and obstacles faced by academic spin-offs and high-technology ventures.

The prevailing issue in this cluster relates to the necessity of including outside members with business experience in teams responsible for managing academic spin-offs. A consensus among researchers has emerged on this topic: the benefits of adding non-scientific members to the team outweigh the potential costs of such transactions (Ben-Hafaïedh et al. 2018; Ferretti et al. 2020; Huynh et al. 2017; Sciarelli et al. 2021; Visintin and Pittino 2014). Teams in academic spin-offs need to develop an entrepreneurial culture to explore and exploit entrepreneurial opportunities to establish a successful long-term business. This can be achieved by expanding the competencies of scientists to the entrepreneurial domain or, which is the case most of the time, by bringing in members to the team that already possess business experience (Lockett et al. 2005; Rasmussen and Wright 2015; Vohora et al. 2004) to more quickly gain venture credibility (Rasmussen et al. 2011) and access to new profitable paths (Vohora et al. 2004).

Patents are another theme present in this cluster. Patents, as a resource, are preceded by entrepreneurial orientation (Walter et al. 2016) and can help entrepreneurial teams obtain higher firm performance (Ferri et al. 2019). In addition, firms that are founded by teams of professors have more patents than firms that are founded by teams of research assistants (Roche et al. 2020).

#### Yellow cluster (4)- finance

Financial topics heavily dominate the yellow cluster. Even though numerous funding sources are available to entrepreneurial teams, only three received noticeable attention from researchers. These include *venture capital*, *business angels*, and *crowdfunding*.

Whether or not the features of entrepreneurial teams play a role in obtaining financing from *venture capitalists* is still a hotly contested debate. On the one hand, researchers have found that instead of general entrepreneurial team characteristics, the prospects of the market in which the firm is operating are the focus of venture capitalists when evaluating business propositions (Hall and Hofer 1993; Zacharakis and Meyer 1998). On the other hand, a different stream of research will argue that some specific attributes of entrepreneurial teams can benefit them in attracting funds from venture capitalists. For instance, ventures that are managed by big teams have a higher probability of getting financed by venture capitalists (Baum and Silverman 2004). Besides team size, the leadership capabilities of at least some team members are significant elements that factor into venture capitalists’ evaluations (Franke et al. 2008). In addition, more experienced teams (Hoenig and Henkel 2015; Hsu 2007; Kolympiris et al. 2018) and teams with more powerful social ties (Hsu 2006; Shane and Stuart 2002) receive higher valuations from venture capitalist firms.

Similar to research on venture capital, authors that analysed the behaviour of *business angels* were interested to find out whether team composition affected evaluations conducted by business angels. The entrepreneurial team’s industry experience and educational background are essential factors in the evaluation process (Becker-Blease and Sohl 2015). Moreover, perceived intangible factors, such as trustworthiness, honesty, the commitment of the team, and intra-team trust, are also vital to business angels (Bammens and Collewaert 2014; Croce et al. 2017; Mason et al. 2017). Lastly, social ties between entrepreneurial teams and business angels affect business angels’ evaluations (Ding et al. 2014).

The third topic of this cluster is related to *crowdfunding*. More similar to an angel than to venture financing, team composition and structure are significant predictors for obtaining funds via crowdfunding platforms. Higher education levels of team members increase funding amounts and the overall success of the crowdfunding campaign (Ahlers et al. 2015; Barbi and Mattioli 2019). Furthermore, bigger entrepreneurial teams are seen by crowd-backers as a positive signal of the venture in the first (Ahlers et al. 2015; Lagazio and Querci 2018; Ralcheva and Roosenboom 2020) and follow-up round of financing (Hornuf et al. 2018).

#### Purple cluster (5)- Transformation

The authors in this cluster have predominantly concerned themselves with transitions and reconfigurations of entrepreneurial teams across the firm’s life-cycle. Various stages and turning points were examined, but the most prominent is the process of *initial public offerings*. Before turning the attention to the initial public offering, it is necessary to point out what entrepreneurial scholars have to say about *changes* that the team and the firm go through in the earlier stages of development.

The importance of initial and later composition of entrepreneurial teams cannot be overstated. Various aspects of members comprising entrepreneurial teams, and the decisions that flow from them, can have immediate and long-lasting effects on organisational structures, functional structures, firm outcomes (Beckman and Burton 2008), and values that are embedded in the firm (Leung et al. 2013). Thus, its members need to plan *changes to the entrepreneurial teams* very meticulously. Teams need to be aware of specific skill sets imposed on their members, which are vital to the firm’s development (Drazin and Kazanjian 1993). If such skills are not present in the current team formations, members need to know how to acquire external members who possess such skill sets (Clarysse et al. 2007; Kor and Misangyi 2008).

These most significant changes to entrepreneurial teams occur at *initial public offerings*. Not all entrepreneurial teams, however, manage their firms to the point at which firms are suitable for initial public offerings. Entrepreneurial teams with more functional diversity that do not lose team members have a higher chance of achieving that goal (Beckman et al. 2007). Even though an initial public offering is a meaningful milestone for entrepreneurial teams, entrepreneurial teams need to be aware of the team-related determinants that factor into the success of an initial public offering. For example, initial public offerings are less likely to fail the higher the tenure of entrepreneurial team members (Fischer and Pollock 2004). In addition, age and functional heterogeneity are also associated with favourable initial public offerings (Xu et al. 2017).

#### Turquoise cluster (6)- internationalisation

The turquoise cluster is saturated with internationalisation research. Entrepreneurial teams are crucial in clarifying the scope of internationalisation activities (Voudouris et al. 2011) and the nature of these activities is shaped by the competencies of team members (Cannone and Ughetto 2014; Hagen and Zucchella 2014). Furthermore, entrepreneurial teams bring various capabilities and resources to the table that solo entrepreneurs cannot provide in matters of internationalisation (Loane et al. 2007). One such resource that predominantly captivated entrepreneurial scholars in this cluster is the entrepreneurial team’s experience.

Even though internationalisation is a function of a diverse set of team experiences (Ganotakis and Love 2012), *international experience* is the prevailing variable in published research. Higher international experience is valuable to entrepreneurial teams since it can lead to better partnership formation and fewer delays in product delivery (Reuber and Fischer 1997). It was also linked to: (i) the employment of more rational processes in the strategic domain (Azam et al. 2018), (ii) the competency to fully capitalise on international opportunities (Park and Rhee 2012), (iii) the ability to effectively overcome the barrier of resource deficiency (Laanti et al. 2007), and (iv) the capacity to exploit the advantages of cluster locations (Fernhaber et al. 2008).

These results are mostly connected to a static view of entrepreneurial teams and internationalisation endeavours. Some authors took a different approach and studied the changes that occurred in firms. It is common to observe a member departing from entrepreneurial teams in the early stages of establishing an international venture. Departures are not necessarily adverse events because they can force the remaining team members to allocate resources more effectively (Loane et al. 2014). Re-evaluating a venture’s position and orientation can also be valuable in cases where specific international customers are lost as well (Crick et al. 2020).

#### Orange cluster (7)- family

The orange cluster is permeated with articles that examine a specific context of entrepreneurial teams in the family entrepreneurship framework. The central focus of researchers in this cluster was to figure out how the *presence of family members* affected the performance of firms that they were responsible for. Numerous insights emerged from these studies. For example, some studies report a harmful effect of high family inclusion in entrepreneurial teams on firm performance (Basco 2013; Kellermanns et al. 2012), while others note the opposite (Pittino et al. 2020). This issue is more complex since a few authors have found that the presence of family members in entrepreneurial teams has a positive effect on firm performance up to a point, after which adding more family members to entrepreneurial teams is inimical to firm performance. In other words, these two variables have an inverted U-shaped relation (Chirico and Bau’, 2014; De Massis et al., 2015). Furthermore, some studies explored the impact of family relations on firm performance more deeply. For example, spousal teams are more equipped to run a firm than teams that incorporate family members that are further apart from them in the family tree (Bird and Zellweger 2018; Brannon et al. 2013).

Before proceeding to the last cluster, it is worthwhile to recognise *entrepreneurial orientation’s* important role in family entrepreneurial teams. Here again, we encounter the presence of non-linear effects. Specifically, the impact of family involvement (Bauweraerts and Colot 2017) and the number of generations present in the team (Sciascia et al. 2013) on entrepreneurial orientation positively affects lower levels, after which the effect becomes negative. Complementary results can be found elsewhere (Cruz and Nordqvist 2012).

#### Brown cluster (8)- community and surroundings

The brown cluster, the smallest one, is the last cluster to be analysed. Topics that are covered by these articles emerged in recent years due to the *changing structure of economic systems* and societal emphasis on *equality* and having a *long-term perspective*.

Entrepreneurial teams need to be knowledgeable about building the team’s reputation in their socio-economically constructed surroundings since the opinions and views of economic actors in such surroundings can determine the legitimacy of their venture (Fisher et al. 2016). Besides just knowing their community, connecting the values of entrepreneurial teams with the values of the community can aid the team in reducing the organisational risk of the venture (Almandoz 2014). Emphasising community needs can also push entrepreneurial teams to better manage *social ventures* in the long run since it is vital for ventures to focus on their social mission from the early stages of development (Battilana et al. 2015).

In addition to social goals, *environmental goals* can emerge through the interactions between entrepreneurial teams and external stakeholders. The design and importance of green goals and related decision-making processes that unwind to achieve these goals are highly firm-specific (Grinevich et al. 2019). The presence of multiple institutional logics is one postulated explanation of this finding. Besides being an essential component of the growth of green ventures, how entrepreneurial teams comprehend institutional logics can profoundly affect the type of entrepreneurial opportunities they pursue and how they pursue them (Dufays and Huybrechts 2016).

Finally, some community failures in achieving *gender equality* in entrepreneurial teams need to be outlined. Women entrepreneurs still struggle to establish leadership roles in entrepreneurial teams (Yang and Aldrich 2014). Furthermore, women’s actions in entrepreneurial teams are more scrutinised than their male counterparts and entrepreneurial teams led by women experience industry-related discrimination (Yang and del Carmen Triana 2019).

## Discussion

To ensure a well-rounded analysis of entrepreneurial teams, this discussion section offers a complementary depiction of the topic through three lenses: (i) research timeline, (ii) theoretical foundations, and (iii) IMO framework. Each lens contains propositions that follow from the obtained results.

### Research timeline

**Fig. 4:**
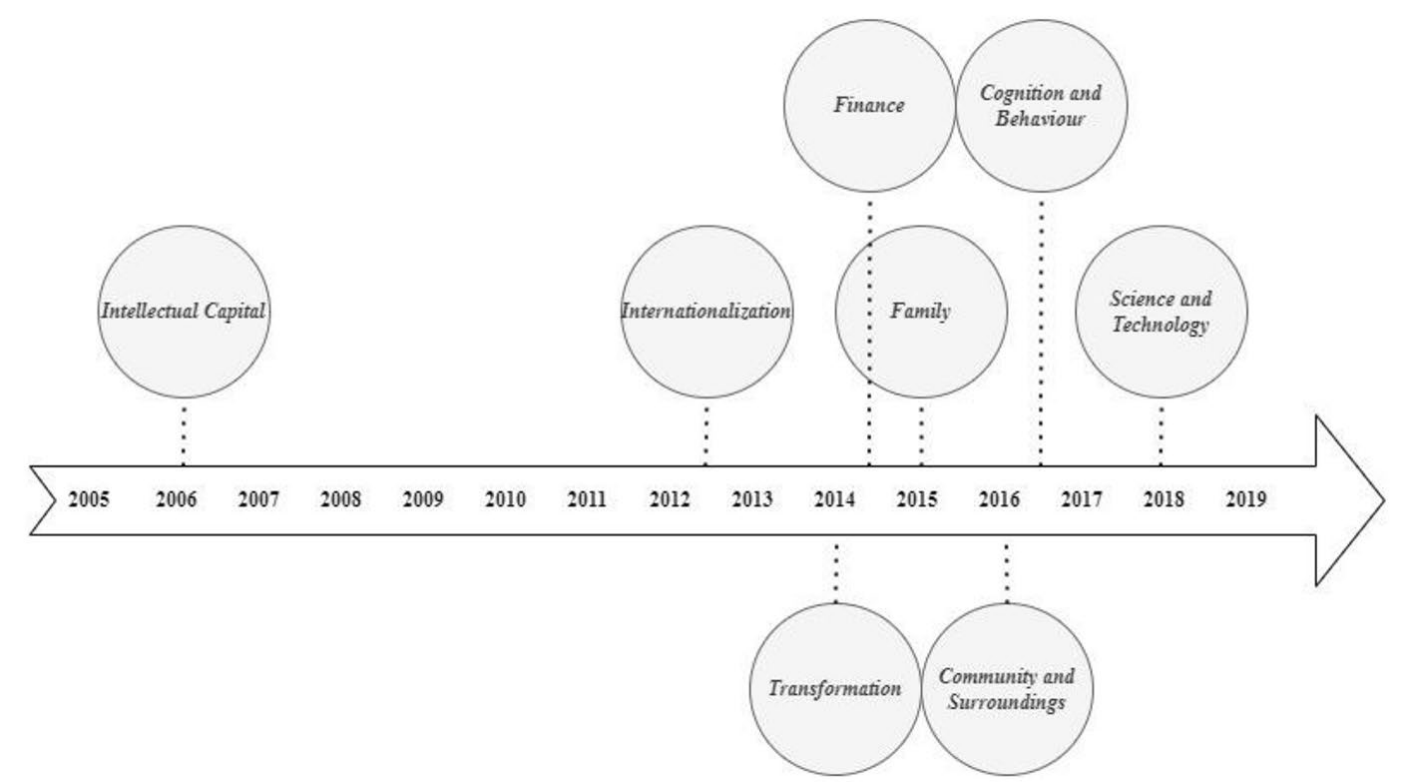
The timeline of published articles Source: compiled by authors

Currently, the literature on entrepreneurial teams does not recognise the contributions across time. Thus, at the moment, there needs to be a better sense of what direction the field is developing in, which subjects were investigated earlier, and which are of more recent interest. A median of all publications in each cluster is calculated. These results are graphically displayed in Fig. 4.

Figure 4 shows that the *Intellectual Capital* cluster is by far the most mature cluster, with a publication median of 2006. This could mean that the *Intellectual Capital* cluster dealt with fundamental issues that inspired the field of entrepreneurial teams. An alternative view is that the issues that were investigated may need to be readdressed with a fresh viewpoint. Next is the *Internationalisation* cluster which is a more recent cluster compared to the *Intellectual Capital* cluster but is lagging when contrasted with the other six clusters. Out of these remaining six clusters, the *Science and Technology* cluster stands out as a bundle of articles interested in novel topics, which given the plethora of publications on academic spin-offs, could be the result of the public emphasis on entrepreneurial universities as catalysts of innovation in the triple helix model (Feola et al. 2021).

One proposition follows from the timeline analysis.

#### Proposition 1

The themes uncovered in the *Internationalisation* cluster and, mainly, in the *Intellectual Capital* cluster are in the most need of revival and a fresh outlook.

### Theoretical foundations

The adapted theoretical foundations are the second lens through which the entrepreneurial teams research is examined. Until now, the only indication of conceptual grounding for undergone studies is found in Klotz et al. (2014), where upper echelons theory was exposed as a central theoretical basis. However, more detailed analyses of theoretical affairs are absent in the literature. Furthermore, no analysis detected how authors conceptualise entrepreneurial teams in thematic groups since it is likely that similar topics use analogous foundational frameworks. In addition, although internally connected, themes are also likely to have relations with other themes in entrepreneurial teams publications. By scrutinising the sampled studies, this section spotlights the most theoretically rich clusters, the latent connections between clusters, underutilised theoretical bases of research, and the implicit consensus of entrepreneurial teams that most scholars agree upon.

Before analysing these frameworks, it is worth pointing out that many articles in each cluster did not explicitly state which over-arching theoretical view they used to support their hypotheses. Even though it is not strictly required for publication and hypothesis justification, this kind of omission slightly reduces the full quality of this study.

**Fig. 5:**
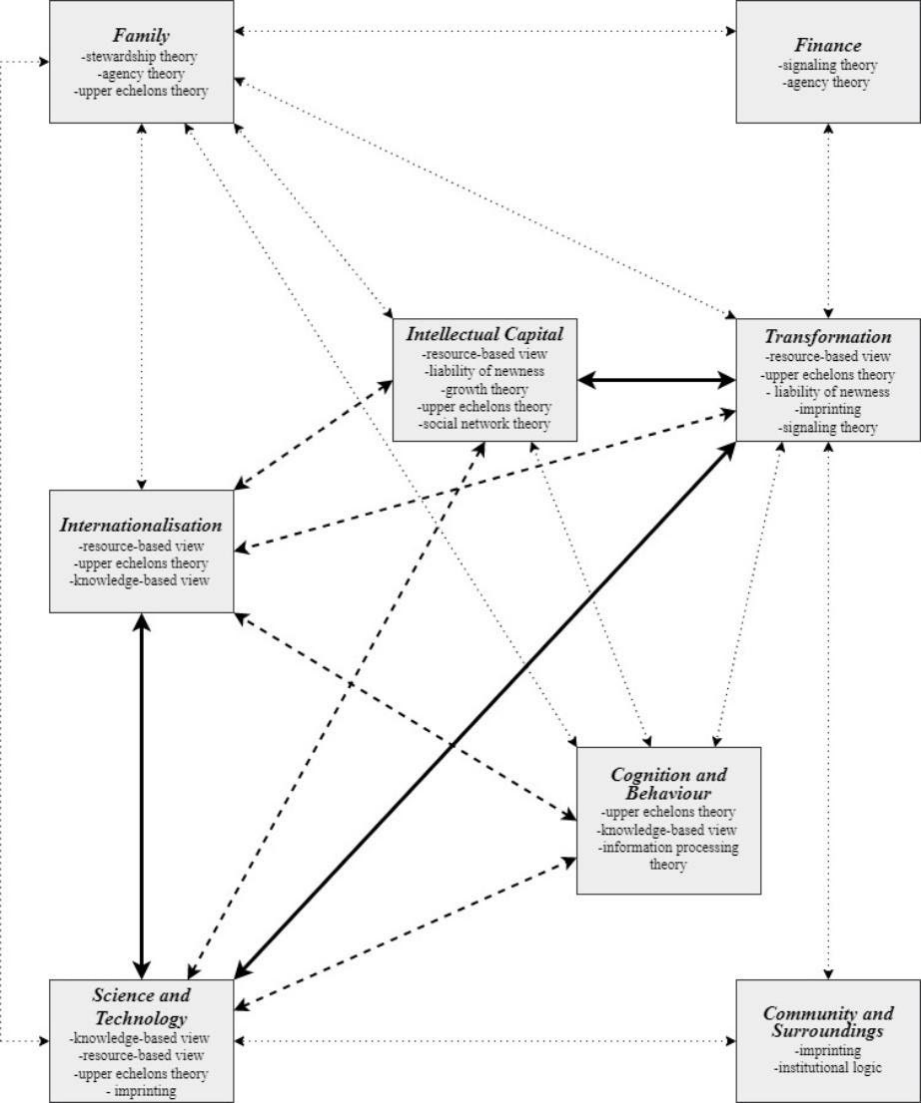
The theoretical foundations of clusters and their connections Source: compiled by authors Notes: clusters that are connected with lines share at least one theoretical framework. Full line means clusters share three theoretical frameworks; dashed line means clusters share two theoretical frameworks; dotted line means clusters share one theoretical framework The frameworks presented are those frameworks that were highly represented in the cluster, meaning the list is not exhaustive

Nonetheless, there is an abundance of utilised theoretical frameworks displayed in Fig. 5.

As was the case nearly a decade ago (Klotz et al. 2014), entrepreneurial teams scholars primarily relied upon the postulates of upper echelons theory in their studies. In fact, the upper echelons theory is utilised in all clusters except the *Finance* and *Community and Surroundings* clusters. This means that entrepreneurial teams are predominantly viewed as groups of people with inherent cognitive limitations whose actions and situational interpretations are a function of various individual attributes (such as their experiences and personalities). These attributes are a dominant force in explaining the observed variations in performances amongst firms (Hambrick and Mason 1984). Unsurprisingly, this theoretical outlook is favoured in the entrepreneurial teams field since it was specifically developed to accompany the intricacies of team functioning.

The second most employed theoretical foundation is the resource-based view. The resource-based view is present in the *Intellectual Capital*, *Transformation*, *Internationalisation*, and *Science and Technology* clusters. This school of thought links the ability of the firm to achieve a competitive advantage with the process of gathering and developing unique resources and capabilities with favourable characteristics. These include a set of diversified tangible and intangible assets, as well as the specific skills that the entrepreneur, or in this case, entrepreneurial teams, possesses (Barney 1991).

The third tier of used theories consists of the knowledge-based view and the imprinting theory. The knowledge-based view is represented in three clusters, the Internationalisation, the *Science and Technology*, and the *Cognition and Behaviour* clusters. This view is expected to be contained in similar clusters to the resource-based view since it can be considered an extension of the resource-based view. However, despite the similarities with the resource-based view, the knowledge-based view has a distinct point of view on resources. More concretely, the knowledge-based view postulates that the most critical resource is the knowledge the entrepreneurs have and that the most important activity that the firm carries out is utilising its knowledge base (Grant 1996). A wholly different approach, but equally represented in research on entrepreneurial teams, is imprinting theory. Imprinting theory states that firms are, at one point during their development, predominantly in the founding phase, highly susceptible to environmental impacts. These impacts produce long-lasting characteristics despite the subsequent environmental changes that produced those characteristics (Marquis and Tilcsik 2013; Stinchcombe 1965). This theory is expressed in the Transformation, the *Science and Technology*, and the *Community and Surroundings* clusters.

The next set of theoretical postulates is utilised in two clusters, which are signalling theory (*Transformation* cluster and *Finance* cluster), agency theory (*Family* cluster and *Finance* cluster) and the liability of newness (*Transformation* cluster and *Intellectual Capital* cluster). Unlike the previously examined theories, these frameworks do not take a broad view of the firm, but they are postulated to explain specific distinct actions that the firm makes. Signalling theory states that entities deliberately send observable and costly signals to other entities in their environment to reduce information asymmetry problems (Spence 1973). The problem of information asymmetry also appears in agency theory, where this problem is tackled from a different angle. Agency theory is interested in issues that arise from interactions between principals and agents. It proposes various monitoring systems, incentives, and contractual obligations to lower the cost stemming from such interactions (Eisenhardt 1989). The last framework in this group is the liability of newness approach to firms. Liability of newness is not a theory per se. However, it is a frame of reference that tries to understand how firms, in their inception stages, overcome inherent limitations due to their size, such as the lack of essential resources (Stinchcombe 1965).

Finally, some theories are contained in just one cluster. These include: (i) stewardship theory (*Family* cluster), (ii) institutional logic (*Community and Surroundings* cluster), (iii) growth theory (*Intellectual Capital* cluster), (iv) social network theory (*Intellectual Capital* cluster), and (v) information processing theory (*Cognition and Behaviour* cluster).

From all of these theoretical examinations, the implied consensus of the majority of scholars on entrepreneurial teams can be deduced. Therefore, the presented commonalities in Proposition 2 could be utilised as a starting point in establishing an *entrepreneurial teams theory*.

#### Proposition 2

Entrepreneurial teams are a group of people where the individual attributes of members and interactions among those members add a valuable and highly enduring contribution to the process of assembling and exploiting key resources, among which knowledge is the most important, to achieve a high level of firm performance. The effects that this process generates are especially relevant in the early stages of the firm’s life-cycle and in situations where information asymmetry problems are present.

Before proceeding to the next section, it is worth looking at the connections between clusters on theoretical grounds. In perspective shown in Fig. 5, the more theoretical grounds are shared between clusters, the more they are connected. Therefore, the most associations are between: (i) *Intellectual Capital* and *Transformation* clusters, (ii) *Transformation* and *Science and Technology* clusters, and (iii) *Science and Technology* and *Internationalisation* clusters. Combining this view with the timeline presented in Fig. 4, it is apparent that the comparative lack of new publications in the *Intellectual Capital* cluster and the *Internationalisation* cluster cannot be attributed to the use of outdated theoretical bases.

Following the information disclosed above, two propositions are put forward.

#### Proposition 3

On the whole, researchers should, from a theoretically foundational viewpoint, strive to better connect the identified eight clusters.

#### Proposition 4

From a theoretical standpoint, the needed rejuvenation of current topics in the *Intellectual Capital* and *Internationalisation* clusters will not come from utilising main frameworks but rather from using auxiliary ones.

### IMO framework

**Fig. 6:**
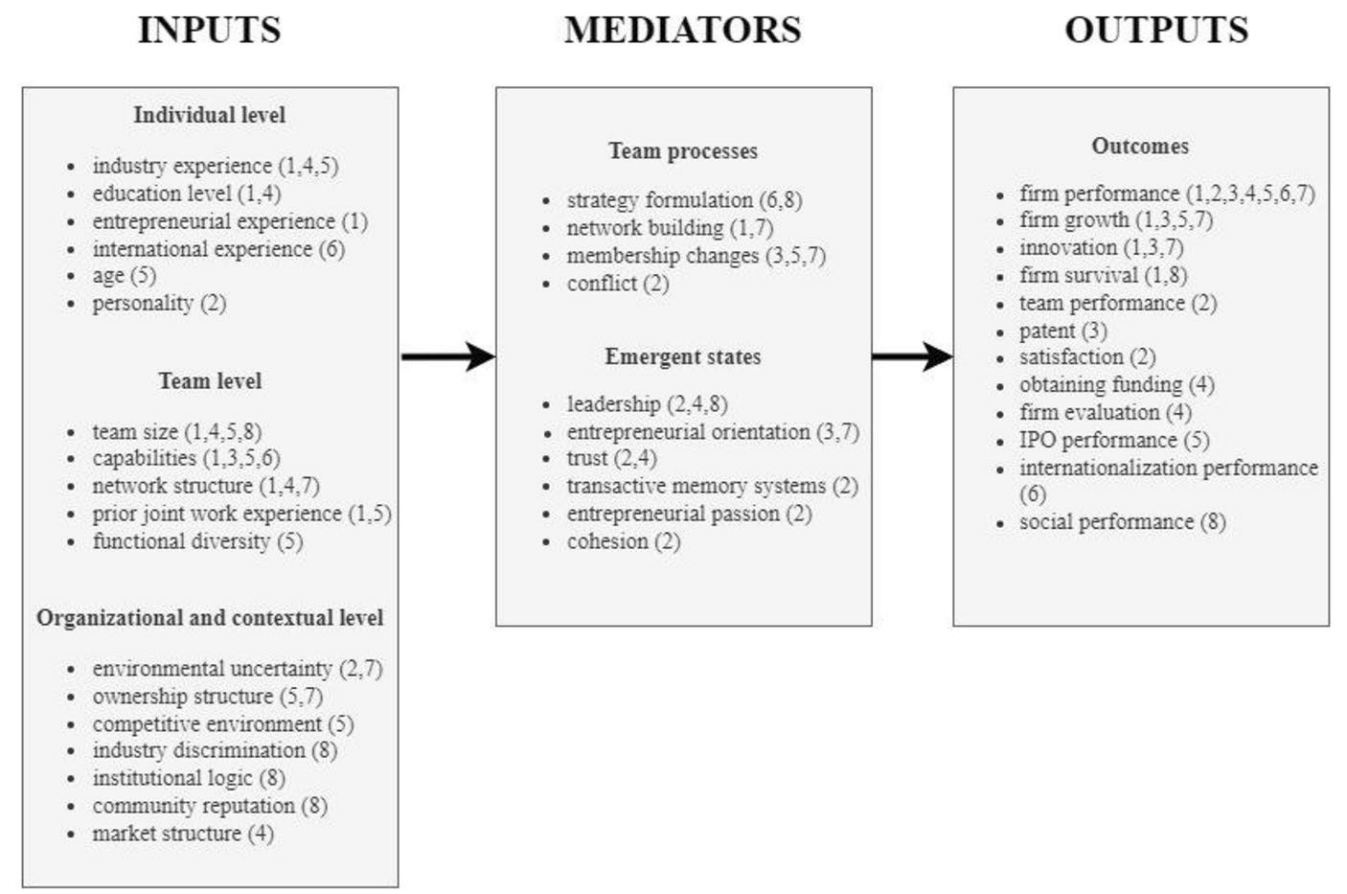
The Input-Mediator-Output framework of influential topics in clusters Source: compiled by authors Notes: the numbers in brackets indicate cluster appearance: 1) *Intellectual Capital* 2) *Cognition and Behaviour* 3) *Science and Technology* 4) *Finance* 5) *Transformation* 6) I*nternationalisation* 7) *Family* 8) *Community and Surroundings* The variables presented are those variables that were highly represented in the cluster, meaning the list is not completely exhaustive

The third approach to discussing the results of the bibliometric analysis is embedded in the IMO framework, shown in Fig. 6. As mentioned previously, Klotz et al. (2014) already adopted the IMO framework for a literature review on entrepreneurial teams. Notwithstanding their immense contribution, this study opts for the same framework but expands it in two ways. Firstly, due to a detailed display of research findings in Sect. 3.2., this study offers a more fine-grained categorisation of input-mediator-output variables. Secondly, on the count that entrepreneurial teams variables are associated with thematic clusters, cluster participation in variable composition and variable placement in the IMO framework can be emphasised.

Before the analysis, it is worth mentioning that the IMO framework can subtly vary depending on the study. The one shown in Fig. 6 follows the approach taken by Klotz et al. (2014). Inputs represent antecedents of the mediating factors, while outputs are the results of mediating factors. Mediators are divided into team processes (actions conducted to achieve a defined goal) and emergent states (space and time-sensitive cognitive and affective states). Most of the variables in Fig. 6 have been elaborated on in Sect. 3.2. Here, the IMO framework is used to identify some ‘global’ characteristics of the field which are not possible in traditional bibliometric analysis.

Figure 6 reveals some interesting insights. Firstly, the individual-level input variables are broad in number but not in cluster diversification. The same applies to the organisational and contextual input variables but does not hold for team-level input variables. Here we see roughly the same number of variables, but they are more dispersed among clusters. Next, regarding mediators, emergent states are more pronounced than team processes. They are, however, highly concentrated in the *Cognition and Behaviour* cluster. When looking at team processes, it is evident that these variables are the least presented in the whole IMO framework. Thirdly, output variables are very broad in sheer number and cluster participation. They exhibit distinct cluster features (such as international performance and patent production). Despite the wide-ranging collection of outcome variables on the firm level, only satisfaction is associated with team-level production. Lastly, the call for future studies by Klotz et al. (2014) to examine the process of strategy formation as a mediator and to consider satisfaction as an output variable has been addressed.

Two propositions are derived from the IMO framework analysis.

#### Proposition 5

Researchers in all thematic clusters, albeit to varying degrees, should put more focus on mediator-level variables.

#### Proposition 6

Considering the output section, researchers in all thematic clusters should focus on team-level variables compared to generic firm performance ones.

All six propositions are presented in Table 8.

**Table 8:** Six propositions that follow from the discussion

Type of analysis	Propositions
*Research timeline*	1. The themes uncovered in the *Internationalisation* cluster and, mainly, in the *Intellectual Capital* cluster are in the most need of revival and a fresh outlook.
*Theoretical foundations*	2. Entrepreneurial teams are a group of people where the individual attributes of members and interactions among those members add a valuable and highly enduring contribution to the process of assembling and exploiting key resources, among which knowledge is the most important, to achieve a high level of firm performance. The effects that this process generates are especially relevant in the early stages of the firm’s life-cycle and in situations where information asymmetry problems are present. 3. On the whole, researchers should, from a theoretically foundational viewpoint, strive to better connect the identified eight clusters. 4. From a theoretical standpoint, the needed rejuvenation of current topics in the *Intellectual Capital* and *Internationalisation* clusters will not come from utilising main frameworks but rather from using auxiliary ones.
*IMO framework*	5. Researchers in all thematic clusters, albeit to varying degrees, should put more focus on mediator-level variables. 6. Considering the output section, researchers in all thematic clusters should focus on team-level variables compared to generic firm performance ones.

Source: compiled by authors

## Agenda for future research

The range of promising areas for future research is vast, given the general comprehensiveness of the field, the number of identified clusters, and the sub-themes that are part of the clusters. The recommendations for future research are based upon the analysis presented in the discussion section and on the overall reactions to the state of the field as reported in the central bibliometric analysis. Future research proposals are made for the entire field of entrepreneurial teams and each identified specific cluster. A summary of possible future pathways, along with recommended references is displayed in Table 9.

**Table 9:** The present and future structure of entrepreneurial teams research

Lines for future research	References
*All clusters* Dynamic capability view Competitive dynamics Process studies Case studies Too-much-of-a-good-thing principle	Teece et al. (1997) Chen and MacMillan (1992); Chen et al. (2007) Jones et al. (2019); Sharma et al. (2022); Steyaert (2007); Zayadin et al. (2022) Eisenhardt and Graebner (2007); Siggelkow (2007) Pierce and Aguinis (2013)
*Intellectual Capital cluster* Structural capital	Chang et al. (2022); Durst and Runar Edvardsson (2012); Manohar Singh and Gupta (2014); Pedro et al. (2018); Turner et al. (2012)
*Cognition and Behaviour cluster* Representational gaps	Cronin and Weingart (2007); Paletz et al. (2013); Pearsall and Venkataramani (2015)
*Science and Technology cluster* Technology adoption	Barnett et al. (2015); Chwolka and Raith (2022); Ding (2011); Kaur et al. (2022); Roberts et al., 2021); Salmony and Kanbach (2022); Zamani (2022)
*Finance cluster* Bootstrapping	Grichnik et al. (2014); Jayawarna et al. (2015); Jonsson and Lindbergh (2013); Neely and Van Auken (2012)
*Transformation cluster* Pivoting	Berends et al. (2021); Kirtley and O’Mahony (2020); Leatherbee and Katila (2020)
*Internationalisation cluster* Risk strategies	Eduardsen and Marinova (2020); Cerrato and Fernhaber (2018); Kiss et al. (2013); Ozkan (2020)
*Family cluster* Succession	Dalpiaz et al. (2014); Daspit et al. (2016); Kubíček and Machek (2019); Sreih et al. (2019)
*Community and Surroundings cluster* Immigrant entrepreneurship	Arrighetti et al. (2014); Dabić et al. (2020b); Kariv et al. (2009)

Source: compiled by authors

### Future research for the entire field

#### Theoretical considerations

On a conceptual note, although the majority of theoretical managerial frameworks have been employed to support the proposed hypotheses, two major frameworks still could be incorporated into the field: (i) the dynamic capability view and (ii) competitive dynamics. These two lines of inquiry are recommended to researchers that prefer to further theoretically expand the field, as opposed to the conceptually unifying approach through the *entrepreneurial teams theory*.

The dynamic capability view (Teece et al., 1997) is a prominent theoretical view in the business management literature and should be incorporated into the entrepreneurial teams field. Future studies could propose more abstract models based on dynamic capability underpinnings and empirically test how the process of resource alignment to the ongoing changes in the environment affects the functioning of entrepreneurial teams and vice versa. In addition, future endeavours could assess how the interplay between team members influences the creation and maintenance of resource-generating and configuring routines.

Competitive dynamics, admittingly not as conceptually developed and represented in the literature as the dynamic capability view, is nonetheless a significant viewpoint for managerial actions. Thus, it could provide a new way of understanding entrepreneurial teams. Specifically, prospective studies could examine how the attributes of entrepreneurial teams change in response to retaliation from competitors in the marketplace (Chen and MacMillan 1992). Furthermore, particular ongoing team processes based on such responses should be suitable for the Awareness-Motivation-Capability framework (Chen et al. 2007).

#### Methodological considerations

In many clusters, a purely quantitative viewpoint is predominant on posed research questions, while a case study approach is far less frequent. This is truer for some clusters than others. For instance, case studies in the *Family* cluster are common, while such a technique is seldom used in the *Cognition and Behaviour* cluster. By opting for a broader spectrum of research designs, authors could portray entrepreneurial teams in a more detailed way, following the guidelines of Eisenhardt and Graebner (2007) and Siggelkow (2007).

Similarly, qualitative studies could devote more attention to the processes that unfold in entrepreneurial teams dynamics and try to apprehend the context in which these processes occur (Jones et al. 2019; Zayadin et al. 2022). As was revealed in the IMO framework, pure team processes are the least researched variables in the field. In the *Finance* cluster, such an approach is almost non-existent. Beyond just the choice of variables, there is a wide range of general process modes available to entrepreneurial scholars (Steyaert 2007) that can broaden the research horizon. Future research could elaborate on one specific process in detail, that being the adjustments firms make in their policies due to interacting with their clients (Sharma et al. 2022).

In addition, future research could test the intricacies of variable relations. Complementing standard linear models, the authors can examine non-linear effects more frequently. In conceptual language terms, they can apply the too-much-of-a-good-thing principle (Pierce and Aguinis 2013). Beyond the contribution that the pure results of such studies have, they can also provide explanations for conflicting findings that have crystallised in a few clusters. Such research is, to a lesser extent, already present, for instance, in the *Family* cluster where quadratic effects are a regular occurrence.

### Future research for each specific cluster

Given that most items of human and relational capital (Pedro et al. 2018) have been utilised as variables in the *Intellectual Capital* cluster, it is unlikely that a significant scientific push in the oldest cluster can be achieved in those areas. However, there is one glaring area that is almost unexplored, and that is structural capital. Structural capital, with human and relational capital, is a critical component of the intellectual capital concept. Yet, the presence of structural capital in the entrepreneurial teams literature, compared to human and relational capital, is barely felt. Consequently, this is a significant research gap in the *Intellectual Capital* cluster. Knowledge management is a prime candidate for future research out of the many components that make up structural capital. How the dynamics of entrepreneurial teams influence every step in the knowledge management process (knowledge identification, creation, retention, transfer, and utilisation) (Durst and Runar Edvardsson 2012) could be a promising pathway forward, as pawed by a recent model demonstrated in the study by Chang et al. (2022). Others could abandon conceptualising teams as an underlying process supporting knowledge management practices (Turner et al. 2012) and instead empirically examine how the knowledge management process is shaped inside entrepreneurial teams (Manohar Singh and Gupta 2014).

Looking at the themes discovered in the *Cognition and Behaviour* cluster, research has only scratched the surface of these broad and interdisciplinary fields. One form of a shared mental model, currently unexplored in an entrepreneurial team setting, is the concept of representational gaps. Representational gaps are present when team members have contradictory approaches to defining and solving a business-related problem, often due to disperse backgrounds of team members (Cronin and Weingart 2007). As a result of such issues, team functioning and performance can be significantly altered. One possible alteration of team functioning worth exploring is the emergence of conflicts based on the persistence of representational gaps (Cronin and Weingart 2007). Therefore, future research can check whether conflicts indeed do come up in entrepreneurial teams that have high representational gaps. If conflicts are present, discerning which sorts of conflict are the outcome of representational gaps is also valuable (Paletz et al. 2013). Others could test whether the negative aspects of representational gaps ultimately prevail and are, thus, detrimental to the performance of team-led entrepreneurial businesses (Pearsall and Venkataramani 2015).

The third cluster, *Science and Technology*, predominantly looked at antecedent factors to innovation performance or firm performance in general. Though such research is highly valuable, the direction of influence, at least in terms of modern technological development, flows in the other direction as well. In other words, firms are not just producers but also recipients of technology (Zamani 2022), a position extremely rarely argued for in the literature on entrepreneurial teams (Ding 2011). One way to advance this argument is to develop a framework where the inner workings of entrepreneurial teams, from a resource assembling stance, are vital in overcoming various barriers to technology adoption (Kaur et al. 2022). A second way could be to incorporate a strand of the technology adoption literature where the identity of individuals is crucial to explain the acceptance of new technologies in firms (Barnett et al. 2015; Roberts et al. 2021). Therefore, well-researched entrepreneurial traits (Salmony and Kanbach 2022), such as overconfidence (Chwolka and Raith 2022), emerging on the team level, could be postulated to be an antecedent for the intent and the act of technology adoption.

Like the *Intellectual Capital* cluster, the *Finance* cluster lacks article representation in one of its key constituents. Namely, the current structure of the *Finance* cluster incorporates many of the most available formal financial instruments for entrepreneurial funding (Drover et al. 2017). However, informal funding opportunities for entrepreneurs are unexplored territory. Accordingly, bootstrapping activities offer auspicious lines of future inquiry. Grichnik et al. (2014) demonstrated a relationship between a team characteristic, namely team size, with the amount of funding through bootstrapping. Future research could thus continue alongside such paths and test whether entrepreneurial teams can overcome the barriers present in bootstrap funding better than single-founded ventures, such as gender composition (Jayawarna et al. 2015) or education levels (Neely and Van Auken 2012). Others could take a different route and postulate that the configuration of entrepreneurial teams plays a significant role in transitioning from bootstrapping financing to more formal financing arrangements (Jonsson and Lindbergh 2013).

The themes in the *Transformation* cluster are related to the changes in team members and the structural changes in the firm. Studies looked at these changes as primarily disconnected themes. However, such a theoretical stance is not necessarily the only vantage point through which transformations can be observed and studied. One candidate for integrating the two sorts of changes is the process of pivoting. Pivoting refers to specific changes where the entrepreneur gradually incorporates strategic additions and retractions following the appearance of new information (Kirtley and O’Mahony, 2020). Using the literature on pivoting, future research could probe into two areas. One is to determine whether or not new team arrivals and/or departures result from tensions between relational and temporal commitments which ultimately commence a pivoting decision (Berends et al. 2021). Secondly, future studies could examine, in more detail, the importance of team characteristics for the whole business process leading up to pivoting. For example, apart from educational background in the probing stage (Leatherbee and Katila 2020), other team attributes might be a factor in explaining the ongoing particularities not only in the probing stage but also in the preceding (hypothesis formulation) and following stage (business idea convergence) culminating in a pivoting event.

Researchers in the *Internationalisation* cluster can develop the cluster by acknowledging that firms put themselves at risk when entering internationalisation activities. Entrepreneurs are primarily aware of such situations and develop strategies accordingly (Eduardsen and Marinova 2020). However, given the idiosyncrasies of firms and the environments they operate and compete in, the chosen paths based on international strategies differ between firms. Adopting the typology of firm internationalisation behaviours related to mitigating risks by Cerrato and Fernhaber (2018), future studies might test the significance of entrepreneurial team-level drivers and shapers of such directionalities in expanding the undertakings of the firm. Secondly, future research could utilise the observation that internationalisation levels depend upon the risk assessments of the entrepreneur (Kiss et al. 2013). When the strategic poster of the firm is not properly adjusted to the risk of the environment where internationalisation is taking place, a process of market retrieval begins (Ozkan 2020). Examining how the capacities of entrepreneurial teams influence the process of a strategic market exit could bring up intriguing findings and initiate a new outlook on the relationship between entrepreneurial teams and internationalisation.

Similar to the *Intellectual Capital* and the *Finance* clusters, one aspect of the *Family* cluster is absent concerning the entrepreneurial teams but is highly represented in the respective field. That aspect is succession. The benefits and issues that stem from succession processes have been looked at for many years (Daspit et al. 2016). Given this observation and the fact that team changes are familiar to entrepreneurial teams scholars, it is surprising to find such a huge vacancy in the literature on entrepreneurial teams. One route future investigations could take is to consider how specific narrative strategies for venture legitimisation during succession (Dalpiaz et al. 2014) affect the functioning and structure of previously composited entrepreneurial teams. A second starting point for future research is to test how the business changes that follow after a successor enters the firm affect the levels of cohesion and conflict in entrepreneurial teams, given that such alterations can significantly change the established way the business was managed (Sreih et al. 2019). Taking a gender perspective (Kubíček and Machek 2019) could also be fruitful in such a framework.

Finally, there are also a few directions that the research in the *Community and Surroundings* cluster can take. First, immigrant entrepreneurship is a topic that is gaining more and more prominence in the literature (Dabić et al. 2020b). Given that these entrepreneurs have different resources, attitudes, and actions, it could be promising to assess to what extent team compositions play a role in exploring and exploiting entrepreneurial opportunities. Specifically, future inquiries could decipher the function of entrepreneurial teams in creating and maintaining specific patterns present in networks of immigrant entrepreneurs (Kariv et al. 2009). Alternatively, scholars could continue the path of Arrighetti et al. (2014) by elaborating on the impetus and sustained momentum in teams of immigrant entrepreneurs that drive the process of breaking out of the enclave structures towards participation in markets outside their communities.

## Conclusion

The main points of this study were to identify key players in entrepreneurial teams research, break down the fundamental themes that ground the entrepreneurial teams field of inquiry, analyse those themes in three ways, and put forward new ways in which the field could develop. This was accomplished using two means of modern bibliometric analysis, performance analysis and scientific mapping, on a sample of 672 indexed articles in the Web of Science database. The utilised performance analysis tools showed the field’s publication dynamics and citation structure. In addition, leading authors, institutions, and papers were identified. From the scientific mapping perspective, eight discrete clusters of research are uncovered, namely *Intellectual Capital*, *Cognition and Behaviour*, *Science and Technology*, *Finance*, *Transformation*, *Internationalisation*, *Family*, and *Community and Surroundings*. These represent the leading topics in the entrepreneurial teams literature. Every cluster was thoroughly examined to detect which ideas most captivated the imagination of scholars. Based upon that investigation, the whole field was inspected through the time-lapse presentation of each cluster, the backdrop of theoretical foundations and connectedness between clusters, and the incorporation of clustering results into the IMO framework. All the findings culminated in the proposed pathways for future research on the level of the entire field and cluster level.

### Theoretical contributions

Theoretical improvements to the field of entrepreneurial teams, arising from tools of science mapping, are aligned with common theoretical implications emanating from bibliometric analysis, as endorsed by Mukherjee et al. (2022). In that sense, the theoretical contributions from this study come in two forms.

Firstly, using objective bibliometric techniques, distinct clusters were discovered that aid researchers in retrieving and communicating existing knowledge patterns. These eight clusters can be viewed as stand-alone fields in their own right. This means that, although links between clusters are present in the form of used theoretical perspectives, the field of entrepreneurial teams is more scattered than previously thought. Therefore, this study upholds the position that the direction of research starts within a cluster and is then encompassed in entrepreneurial teams research, not the other way around. Consequently, research on entrepreneurial teams lacks a conceptually coherent and uniting framework that accommodates all clusters. Because of this absence, research questions related to entrepreneurial teams have to be integrated from extant economics fields. By presenting a purely theoretical common ground of the conducted research so far, this study asserted an implied scholarly consensus on the conception of entrepreneurial teams. Scholars could employ this understanding of entrepreneurial teams as a foundation to build a fully independent framework, here labelled as an *entrepreneurial teams theory*. *Entrepreneurial teams theory*, as expressed in this study, does not contradict the definition of entrepreneurial teams proposed by Knight et al. (2020) since it was the definition used to gather the articles from which the consensus was identified. Rather, the *entrepreneurial teams theory* could provide distinct theoretical support for a practically applicable definition of entrepreneurial teams. The advancement of such a theory is warranted for the progress of the entire field since it was shown that upper echelons theory, a predominant framework used by researchers, did not fully accommodate the intricacies and attributes of entrepreneurial teams.

Secondly, this study identified numerous gaps in our understanding of entrepreneurial teams functioning through the analysis of cluster-specific detailed findings and trend analysis. Setting aside cluster-related idiosyncrasies, the overall verdict is that researchers still have not utilised all the major research streams at their disposal. While this inference should not be surprising in some clusters, it is especially unexpected in the *Intellectual Capital* cluster, given the historical starting point of research and the breadth of subject matters under its umbrella. Therefore, this study also highlights the need for scientists to explore the topics highly present in each cluster individually and integrate them into a broader entrepreneurial teams research umbrella.

### Practical contributions

There are also practical contributions revealed in a bibliometric analysis (Mukherjee et al. 2022). Here they are expressed in two ways.

The first practical contribution applies to scientific research conduct. The analysis from the results obtained in the performance analysis section of this study demonstrate no significant biases on the institutional, publication, and author levels. However, article production is predominantly related to developed economies, especially to the United States of America, which, at the moment, cannot be explained simply in terms of demographics. Consequently, this study alludes to the existence of geographical bias in current research. On the other hand, more contextually rich studies are required before making broad and globally-applicable factual statements from the empirical findings.

The second line of practical contributions pertains to managerial and entrepreneurial applications of the findings. Continuing along the uncovered geographical bias, entrepreneurs who manage their firms in teams in developed countries benefit more from the sampled results, given that their context is accounted for. The opposite holds for entrepreneurial teams in developing countries. They must approach the empirical results more cautiously since the discovered relations might be altered in their socio-economical setting. Although worries exist in terms of context, for the most part, they should not exist in terms of the timeline of published studies. In that respect, the *Intellectual Capital* cluster is the only troublesome cluster. Real-world entrepreneurs should be alert that the general findings connected to the *Intellectual Capital* cluster might not be applicable to their current situation, given that 2006 is the publication median for that cluster.

### Limitations

Naturally, this study has some limitations. Firstly, only one scientific database where articles are indexed was used. This could limit the breadth of topics that are described in this paper. The second limitation is connected to the problem of defining entrepreneurial teams. As mentioned in the introduction, many synonyms of entrepreneurial teams are interchangeably applied in the literature. Even though a broad spectrum of synonyms was employed in consonance with previous literature reviews, there is the possibility that some articles were not identified if the authors used a different set of words to characterise their research agenda. The final limitation is inherent in almost all literature reviews, which is the subjective nature of authors’ decisions in the article inclusion process. Albeit the bibliometric analysis is less prone to this issue, it is still present in studies that use bibliometric tools to analyse a field. This concern is manifested in this study in the form of the uncertainty that constitutes a suitable entrepreneurial teams article. In other words, there is a probability that some articles were excluded from this study because the sample details were not disclosed in great detail. Decisions of this kind have to be made based on the authors’ judgements rather than using unbiased quantitative apparatuses.

Despite these limitations, the authors are confident that this article will inspire fellow scientists in selecting their directions while investigating features of entrepreneurial teams. We look forward to seeing the further development of the field in the future.
